# Harnessing Probiotics to Combat Candidiasis: Mechanisms, Evidence, and Future Directions

**DOI:** 10.3390/jof11110779

**Published:** 2025-10-29

**Authors:** Emma Wright, Nisha Valand, Umakhanth Venkatraman Girija

**Affiliations:** Leicester School of Allied Health Sciences, Faculty of Health & Life Sciences, De Montfort University, Leicester LE1 9BH, UK

**Keywords:** probiotics, *Candida*, antifungal

## Abstract

*Candida* species are common commensals within the human microbiome but can transition opportunistically to pathogenic states when host–microbe homeostasis is disrupted. Their ability to adhere to mucosa and implanted medical devices, form thick biofilms, and invade epithelial tissues makes candidiasis particularly harmful in immunocompromised and elderly populations. This review examines the reported antifungal activity of common probiotic genera such as *Lactobacillus*, *Bacillus*, *Bifidobacterium*, and *Saccharomyces* across the oral cavity, gastrointestinal tract, and vaginal tract. The probiotic mechanisms of action, such as competitive exclusion, secretion of antifungal metabolites, and immunomodulation, are explored in detail, and research methodologies are scrutinised to assess the robustness of current evidence. This review compiles evidence from a variety of studies and clinical trials showing certain probiotic strains and formulations have the ability to significantly decrease *Candida* colonisation and reduce candidiasis symptom prevalence. Although outcomes vary greatly between probiotic strains tested, species of *Candida* targeted, and specific site of infection, it is clear that selected probiotic species and their secreted substances can have prominent anti-*Candida* effects and promote tangible clinical improvements. Future directions for the field of probiotic study are suggested, including the roles of prebiotics, postbiotics, and synbiotic formulations to enhance probiotic efficacy against candidiasis.

## 1. Introduction

*Candida* is a fungal genus consisting of around 200 species and is the most common cause of fungal infections worldwide [1]. *Candida* has had a symbiotic relationship with humans for thousands of years and is estimated to be present in 40–60% of the population [2,3]. *Candida* thrives in warm, moist environments [4], typically colonising the mouth, gastrointestinal tract, skin, and vaginal mucosa [5]. However, *Candida* is an opportunistic pathogen [6], meaning dysbiosis can result in overgrowth and infection (candidiasis), transitioning its relationship with the human body from symbiotic to pathogenic [7]. It is a phenotypically and morphologically plastic microorganism, capable of adapting to diverse host environments, such as steep oxygen gradients ranging from anaerobic to ~21% oxygen [8]. Candidiasis can be divided into superficial and invasive infections [9]. Superficial candidiasis involves the skin or mucous membranes, causing conditions such as oral or vaginal thrush, and is treated with topical antifungals. Invasive candidiasis is a life-threatening, systemic infection whereby the fungus enters the bloodstream (candidaemia) or infects organs, causing deep-seated abscesses requiring intravenous or oral antifungal medications [10]. Invasive infection of the peritoneal cavity by *Candida* is a recognised but fairly uncommon complication of abdominal surgery or peritoneal dialysis arising from several risk factors of surgical intervention such as disruption of mucosal barriers, contamination, and prolonged use of broad-spectrum antibiotics [11].

*Candida*’s opportunistic nature means it is capable of infecting virtually any part of the body. Symptoms of candidiasis depend on the area of the body affected. Oral candidiasis (commonly referred to as oral thrush) presents as a white coating on the tongue, causing an unpleasant taste and sore mouth for the patient [12]. Vaginal thrush causes intense itching and irritation in and around the vagina, accompanied by a characteristic clumpy white discharge, often said to resemble cottage cheese [13]. Candidiasis of the gut is more difficult to diagnose, as symptoms are broad and non-specific, such as abdominal pain, diarrhoea, fatigue, fever, and mucus discharge in stool [14]. While infections typically involve mucosal or superficial sites, other documented sites of invasive infection include the eyes [15], bones [16], and lungs [17]—often occurring in immunocompromised or critically ill patients. *Candida albicans* accounts for around 50% of all *Candida* infections, with non-*albicans* species such as *Candida tropicalis* and *Candida glabrata* each accounting for 15–20% of cases [18]. Common antifungal treatments for candidiasis may be topical (Clotrimazole), oral (Fluconazole), or intravenous (Caspofungin), depending on the location, severity, and species of *Candida* infection [19]. Antifungal drugs are classified into three major classes: azoles, polyenes, and echinocandins, each with distinct mechanisms of action. Azoles inhibit ergosterol synthesis, polyenes form lysis-causing pores, and echinocandins inhibit β-1,3-glucan synthesis, compromising fungal cell wall integrity [20].

There are an estimated 3.8 million deaths from fungal infections per year—double that of a decade ago [21]. A total of 90% of these deaths are attributed to just four fungal genera: *Candida*, *Aspergillus*, *Cryptococcus*, and *Pneumocystis* [22]. However, precise data on *Candida*’s specific contribution to this figure remain limited, as fungal infections are frequently underdiagnosed and not consistently reported to public health authorities, such as the Centre for Disease control and Prevention. In part, the figure of 3.8 million deaths can be attributed to a concerning rise in antifungal resistance observed by the World Health Organisation, with some *Candida* spp. reported to be pan-resistant to all three major classes of antifungals [23]. Candidiasis can be particularly harmful for immunocompromised individuals, such as those with HIV/AIDS, or those undergoing chemotherapy or lifelong immunosuppressant medication following organ transplantation [24]. *Candida* is, therefore, a major contributor to nosocomial infections, given the common use of broad-spectrum antibiotics in healthcare settings [25] that may disrupt microbial balance [26]. Frequent catheterisation and surgical procedures provide *Candida* with direct access to deep tissues and the bloodstream, resulting in acute and invasive candidiasis [27]. Candidaemia is the most common manifestation of invasive candidiasis, affecting over 250,000 people globally each year, and responsible for more than 50,000 deaths annually [28]. Candidaemia mortality rates lie between 32% and 66% [29], depending on geographical location and causative species, even when patients receive antifungal treatment [30]. A key factor in the increasing prevalence of fatal candidiasis is a recent global shift towards non-*albicans* species, particularly *Candida auris*, which has been reported in five continents to date [31]. *C. auris* is known to persist on surfaces and medical equipment, meaning it spreads easily and rapidly in healthcare environments, especially intensive care units with critically ill or immunocompromised patients, such as the elderly and neonates [28]. Moreover, *C. auris* is multidrug resistant to all three major classes of antifungals (azoles, echinocandins, and polyenes), underscoring the importance of developing alternative treatments [32].

According to the WHO, the development of new antifungal drugs remains limited due to the difficulty of identifying novel targets, the lengthy development process, and a typically low return on investment [33]. The limited availability of novel antifungal drugs, therefore, necessitates research into alternative or adjunct therapies. The global shift towards more virulent and less treatable species such as *C. auris* means effective prophylactic or curative treatments for candidiasis are more urgent than ever, prompting increased research into immunotherapies [34] and probiotics. Whilst various adjunctive treatments such as immunotherapy, vaccines, and combination therapies have shown promise in managing candidiasis, this review focuses on probiotics due to their affordability, accessibility, and safety profile. Furthermore, unlike immunotherapies or vaccines, probiotics offer a practical and sustainable approach that can be incorporated into preventive or therapeutic strategies without the complex manufacturing and regulatory requirements often associated with immunotherapeutic interventions. Probiotics are live microorganisms that confer health benefits upon the host, such as improving microbiome diversity, strengthening the gut barrier, and improving general immune health [35]. Probiotics feed upon indigestible nutrients in the diet, such as fibre and oligosaccharides, suggesting that a varied and balanced diet is important in promoting a healthy gut microbiome [36]. Fermentation of such prebiotics by probiotics produces metabolic byproducts known as postbiotics, many of which are suggested to be the functional compounds responsible for probiotics’ beneficial effects on the host [37,38]. Investigating strains of interest and ascertaining the true functional compounds eliciting anti-*Candida* effects may be key in developing novel candidiasis treatments.

## 2. Mechanisms of *Candida* Virulence, Resistance, and Immune Cross-Talk

*Candida* invades the human immune system in a number of ways, including tissue adhesion, biofilm formation, phenotypic switching, and drug-resistant mutations [39]. Tissue adhesion is essential for candidal colonisation, as it prevents physical clearance and allows biofilm formation.

*Candida* utilises adhesion proteins, such as agglutinin-like sequence (ALS) and epithelial adhesion protein 1 (EAP1), to bind to host epithelial cells by recognising molecules such as laminin and fibronectin [40]. *Candida* can then secrete hydrolytic enzymes to penetrate deep into tissue and anchor the infection. Tissue adhesion is a vital precursor to the formation of biofilms, a structured ecosystem of *Candida* cells embedded within a matrix of extracellular polymeric substances (EPS), such as polysaccharides, proteins, and lipids [41]. Although *C. albicans* has long been the most frequently isolated species from intensive care units (ICUs), the non-*albicans* species *Candida parapsilosis* is the second most commonly isolated species in ICUs due to its ability to form biofilms on internal medical devices and catheters [32,42]. The components of EPS ensure a stable 3D architecture whilst trapping nutrients to allow candidal survival and growth. Thick biofilms are particularly resilient to antifungal drugs, and their presence on implanted medical devices, catheters, and dentures means invasive and drug-resistant infections frequently arise in those who are critically ill or elderly [43].

Following tissue adhesion, *Candida* has the ability to perform phenotypic switching, a reversible epigenetic transition from one distinct cellular state to another [44]. The most notable example of this is the white–opaque switch. White fungal cells are round, smooth, and have higher virulence than flat elongated opaque cells. The relevance of this epigenetic switch is its ability to adapt the fungi to differing host niches. White cells thrive in bloodstream infections and are less easily phagocytosed, whereas opaque cells are best suited to skin infections [45]. Phenotypic switching is, therefore, a vital survival and reproductive mechanism allowing for the pathogenicity of *Candida*. Epigenetic switches are further empowered by morphological switches, such as from the yeast to hyphal form. *Candida* yeast is made up of round, budding cells that can easily disseminate around the body, suitable for bloodstream infections. When transitioning to hyphae, these cells form long, filamentous structures suitable for tissue invasion and biofilm formation, initiating drug-resistant, deep-rooted infection [46]. Phenotypic and morphological switching can be triggered by a variety of factors such as pH, temperature, CO_2_ levels, and stress conditions [47]. The reversible nature of phenotypic switching ensures *Candida* is able to adapt to host niches and evade immune responses as and when necessary. The careful control of this process is mediated via the sophisticated communicative system of quorum sensing, whereby fungal cells can coordinate behaviour [48]. This is a density-dependent signalling method, where cells release quorum sensing molecules called autoinducers (AIs). Once a threshold concentration of AIs is attained, gene expression changes are triggered across the colony [49]. Farnesol is a key AI, particularly in *C. albicans*, with the ability to suppress hyphal growth and biofilm formation [50]. This regulatory role allows yeast-form dominance, which favours dissemination and spread of infection [51]. Limiting filamentous hyphal growth conserves nutrients in the colony microenvironment, ensuring its longevity.

Drug resistance has a large part to play in the number of invasive and treatment-resistant *Candida* infections. Several *Candida* species often exhibit mutations of ergosterol synthase encoding gene (*ERG11*), preventing azoles from targeting the enzyme responsible for ergosterol synthesis [52]. Unable to weaken the fungal membrane and cause cell lysis, the drug is rendered ineffective. In *C. glabrata*, mutations have developed in order to code for an overexpression of membrane transporters, such as efflux pumps, a key azole resistance mechanism [53]. Interestingly, *Candida krusei* is resistant to azoles, not through mutation but due to the presence of naturally occurring efflux pumps. *Candida* uses the dietary mineral iron in order to grow and cause infection [54]. The body sequesters iron in proteins such as transferrin and ferritin in an effort to prevent nutritional immunity; however, *C. albicans* has evolved iron acquisition systems to extract iron from host proteins, a process thought to contribute to its multidrug resistance.

In healthy individuals, *Candida* is eliminated through both innate and adaptive immunity. Immune cells recognise cell wall components, such as chitin, β1-3 glucans, and β1-6 glucans, via pathogen-associated molecular pattern (PAMP)–pattern recognition receptor (PRR) interactions, triggering phagocytosis and activating immune signalling cascades that produce cytokines, chemokines, reactive oxygen species, and neutrophil extracellular traps [55,56]. Cell wall components also interact with Toll-like receptors (TLRs) and C-type lectin receptors (CLRs), such as Dectin-1, inducing the secretion of IL-10, a key anti-inflammatory cytokine [57]. The immune system distinguishes fungal morphology: yeast forms induce IL-12 and a Th1 response, while hyphal forms stimulate IL-4 and a Th2 response [58]. The complement system contributes to clearance via the classical, lectin, and alternative pathways [59]. Naturally occurring anti-*Candida* antibodies aid opsonisation, inhibit adhesion and growth, and neutralise fungal enzymes [60]. Clearance relies on dendritic cells, macrophages, and neutrophils. Dendritic cells patrol mucosal tissues, present antigens to T-cells, and sense yeast and hyphal forms [61]. Macrophages engulf fungi, although some species can survive or replicate intracellularly [62]. Neutrophils, recruited by chemokines from epithelial cells and macrophages, use CLRs, complement receptor-3, and occasionally TLRs to phagocytose fungal cells [63,64]. Several *Candida* species secrete aspartyl proteases that degrade complement proteins (C3, C4, C5) and lectin pathway components, reducing complement activation. Secreted enzymes also inactivate antimicrobial peptides and disrupt pattern recognition receptors, impairing immune detection and killing [59,61,65]. Additionally, some species recruit host complement regulators, such as factor H and C4b-binding protein, via surface or secreted proteins like pH related antigen-1, helping them evade complement-mediated clearance [66,67].

**Figure 1:**
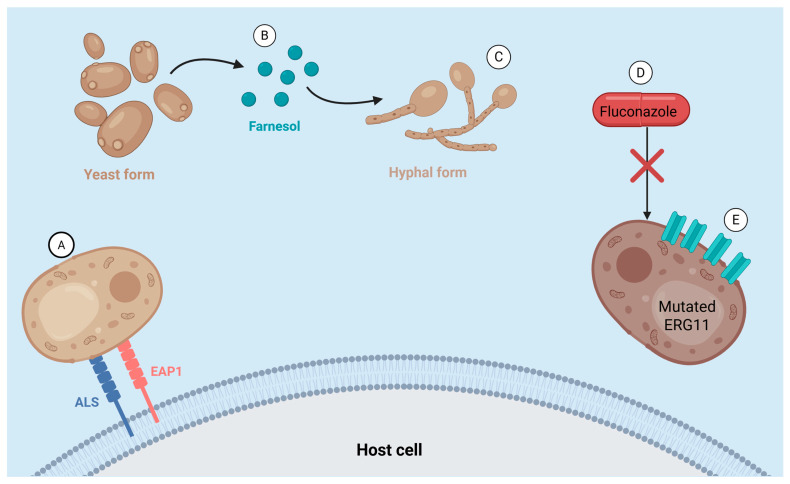
Pathogenic mechanisms and drug resistance strategies of *Candida*. (**A**) *Candida* uses adhesion proteins ALS and EAP1 to adhere to host cells and initiate infection. (**B**) When autoinducer molecules such as farnesol reach threshold concentration, quorum sensing is triggered, a type of signalling allowing coordinated, colony-wide action such as phenotypic switching. (**C**) Phenotypic switching from yeast form (buds) to hyphal form (filamentous), allowing biofilm formation and tissue penetration for invasive infection. (**D**) Azoles are ineffective against *Candida* strains with ERG11 mutation, as they can no longer target lanosterol 14α-demethylase. (**E**) *C. glabrata* often incurs mutations causing an overexpression of efflux pumps, and *C. krusei* naturally exhibits this property, meaning azoles are actively expelled and lose efficacy. Created in BioRender. Wright, E. (2025) https://BioRender.com/x4poaju (accessed on 14 September 2025).

*Candida*’s ability to evade both the human immune system and antifungal drug treatments (Figure 1) is making it increasingly difficult to treat, highlighting the importance of proactive prevention; however, the extent to which probiotics may hold the answer to this need is controversial.

## 3. Probiotics—Introduction and Mechanisms of Action

Gut dysbiosis or an impaired intestinal barrier is a major predisposing factor for invasive *C. albicans* infections that colonise the intestines and translocate through the intestinal wall [68], underscoring the importance of an abundant and balanced gut microbiome. Probiotics are live, non-pathogenic bacteria that are usually administered as an oral tablet or liquid drink, with the aim of increasing the number and biodiversity of gut microflora [69]. The gut microbiome generally consists of four key bacterial phyla, making up 99% of all gut microbes. These are Firmicutes, Bacteroidetes, Actinobacteria, and Proteobacteria [70]. A healthy gut should also be colonised with fungi such as *Candida*, *Saccharomyces*, and *Malassezia* as well as viruses such as bacteriophages [71,72]. Achieving a healthy balance and regulation of the microbiota is essential for overall gut health.

The word probiotic comes from Greek “pro bios”, which means “for life” [73], and was first coined scientifically in 1954 by Ferdinand Vergin and later defined by Daniel Lilly and Rosalie Stillwell in 1965 [74]. Probiotics evade mechanical clearance by adhering to host epithelial surfaces via surface adhesins that specifically recognise transmembrane proteins, including integrins and cadherins [75]. Studies suggest that a greater number and diversity of commensal gut bacteria strengthen the intestinal barrier and have immunomodulatory effects, such as suppressing pro-inflammatory cytokines [76]. Research indicates that certain probiotic strains such as *Lactobacillus* can suppress *Candida* growth and biofilm formation [77]. Using probiotics alongside antifungal drugs has been shown to enhance the rate of clinical cure and reduce relapse rates [78], making probiotic supplementation a promising novel adjunct therapy for both infection cure and recurrence prevention.

Globalisation has caused an increase in ‘western diets’ high in fat, sugar, and carbohydrates as well as increased alcohol intake [79]. Diets high in carbohydrates have been identified as a risk factor for gut candidiasis, as these are metabolised by *Candida* spp., fermenting them into starches and sugars that fuel fungal growth and can trigger a shift from a commensal to pathogenic form [80]. High alcohol consumption is another risk factor for gut candidiasis, as many alcoholic beverages contain sugars that act as food sources for *Candida* [81]. Alcohol is a well-established contributor to gut dysbiosis, as it disrupts the balance of the intestinal microbiome, impairs immune function, and creates an environment that allows opportunistic pathogens such as *Candida* to proliferate [82]. Recognising the dietary components contributing to an increased risk of gut candidiasis is essential, as it highlights the limitations of probiotic therapy when used in isolation. Further research is required to determine whether integrating dietary modifications—particularly reducing the intake of sugars, carbohydrates, and alcohol—alongside probiotic treatment could enhance therapeutic efficacy.

Probiotics generally consist of one or more five key genera: *Lactobacillus*, *Bacillus*, *Bifidobacterium*, *Streptococcus*, and *Saccharomyces* [83]. The number and identity of strains within a probiotic mixture can vary widely, dependent on the aim of the product. Single strain probiotics may be targeted at alleviating a specific condition, such as *Lacticaseibacillus rhamnosus* for antibiotic-associated diarrhoea [84]. Multi-strain probiotics (containing two to five strains) are usually aimed at achieving general gut health or immune improvements. Broad-spectrum probiotics often contain over 20 different strains of bacteria and are marketed as comprehensive microbiome support. Multiple probiotic strains may work synergistically to improve overall health; however, this balance must be carefully formulated, as not all strains are compatible [85]. Clinical evidence of compatible strains is important to ensure the safety and efficacy of a mixture. A key example of the potential dangers of probiotics was reported by scientists at Osaka University, Japan, in 2024. There were five cases of *Clostridium butyricum* bacteraemia in patients who had been taking probiotics containing *C. butyricum*. One of these cases, a 70-year-old male, was fatal. Whilst most of these patients were immunocompromised, the findings highlight the potential risk that probiotics may have on a small subset of the population and underscore the need for careful clinical judgement when considering their use [86].

Numerous mechanisms of probiotic action have been elucidated (Table 1), ranging from simple physical blockades and competitive exclusion to more complex modulation of the gut microbiome and synthesis of neurotransmitters [87]. Several well-studied *Lactobacillus* and *Bifidobacterium* species produce surface proteins aiding adhesion to host tissues, such as lipoteichoic acids and exopolysaccharides [88]. Probiotic binding to epithelial surfaces often occurs in locations that pathogenic bacteria or fungi may otherwise exploit, creating a physical barrier that prevents pathogens, such as *Escherichia coli* or *Salmonella spp.*, from establishing infection [89]. These probiotics also consume local nutrients, depriving pathogens of essential growth substrates [90]. *Lactobacillus* species, such as *Lactobacillus bulgaricus*, are particularly rapid at metabolising simple sugars, in turn acidifying the microenvironment and limiting pathogen viability [91]. *Lactobacillus* and *Bifidobacterium* species are known to produce bacteriocins, such as nisin and plantaricin, peptides that inhibit or selectively kill competing microbes [92]. The ability of certain probiotic strains to inhibit and eliminate pathogenic microbes underpins their potential as prophylactic antifungal agents.

The probiotic strains *Bifidobacterium bifidum* and *Limosilactobacillus reuteri* use surface receptor mimicry to prevent pathogen interaction with the epithelial membrane [93]. Pathogenic fungi, such as *C. albicans*, initiate infection by binding to glycoproteins or glycolipids on host cells [94]. *B. bifidum* and *L. reuteri* are able to express molecules mimicking these receptors and act as decoys to prevent actual pathogen–epithelium binding. Probiotics are also able to interfere with the quorum sensing of pathogenic fungal infections that have already taken hold by interfering with AIs and thereby preventing them from reaching the critical threshold concentration required for density-dependent fungal communication [95]. This interference with quorum sensing is called quorum quenching and is a key mechanism utilised in probiotic formulations [96]. *Bacillus* spp. are particularly rich in lactonase genes, facilitating the degradation of AIs, thus preventing threshold concentration from being reached [97]. Destruction of AIs is not the only method of quorum quenching; some bacteria also produce metabolites that competitively bind to quorum sensing receptors without activating them in a form of receptor antagonism [98,99]. Autoinducer mimicry and quorum sensing sabotage are some of the most nuanced and underexplored aspects of probiotic–pathogen interactions. Only a small number of strains such as *Bacillus subtilis* have been evidenced to mimic AIs, but this powerful ability can lead to premature activation of quorum sensing, virulence dysregulation, and biofilm destabilisation, making these probiotics potentially very effective at targeting *Candida* infections [100].

**Table 1:** Mechanisms of probiotic action against the opportunistic pathogen *Candida*.

Mechanism of Probiotic Action	Example Strains	Anti-*Candida* Effect	References
Physical barrier via adhesion	*Lactobacillus* spp., *Bifidobacterium* spp.	Adhesion to epithelial surfaces physically inhibits *Candida* from binding and initiating infection	[88,89]
Nutrient competition	*L. bulgaricus*	Probiotic colonisation depletes nutrients, creating scarcity for *Candida*	[90,91]
Surface receptor mimicry	*Bifidobacterium bifidum*, *Limosilactobacillus reuteri*	Mimics host glycoproteins or glycolipids to act as decoys, preventing *Candida* binding to host epithelial cells	[93,94]
Quorum quenching	*Bacillus subtilis*	Degrades autoinducers or blocks receptors to disrupt *Candida* colony communication, virulence, and biofilm stability	[95,100]
Hydrogen peroxide production	*L. reuteri*	Activates MAPK, EGFR, and NK-κB immune signalling pathways, enhances epithelial defences, and induces oxidative stress in *Candida*	[101,102]
Lactic acid production	*Lactobacillus acidophilus*, *L. rhamnosus*, *B. bifidum*, and *Leuconostoc mesenteroides*	Acidifies the environment, inhibiting growth and filamentation of *Candida*, suppressing its virulence. Also remodels *Candida*’s cell wall to expose β-glucan and chitin to the immune system for easier recognition	[103]
Biosurfactant production	*Limosilactobacillus fermentum*, *L. acidophilus*, *Lactobacillus paracasei*, *Lactiplantibacillus plantarum*, *Lactococcus lactis*, *Streptococcus thermophilus*, *Propionibacterium freudenreichii*, and *Levilactobacillus brevis* CV8LAC	Reduces *Candida* adhesion and biofilm formation on surfaces such as medical grade silicone by up to 90%	[104,105]
Short chain fatty acid production	*Clostridium butyricum* CBM 588, *Roseburia intestinalis*	Butyrate strengthens the epithelial barrier via tight junctions and supports colonocyte energy needs, protecting against *Candida* colonisation	[106,107,108]
Reuterin production	*L. reuteri* ATCC PTA 6475, ATCC PTA 5289, ATCC PTA 55730, and CF48-3A	3-HPA and acrolein disrupt *Candida* via oxidative stress and enzymatic inhibition	[109,110,111]
Bacteriocin production	*L. lactis*, *Lactobacillus* spp., *Bifidobacterium* spp.	Nisin disrupts *Candida* biofilm formation and enhances membrane permeability. Also enhances antifungal drug efficacy by lowering the minimum inhibitory concentration (MIC)	[112,113,114]
General anti-biofilm activity	*L. acidophilus* ATCC 4356, *L. brevis* CV8LAC, and *L. lactis*	Inhibits biofilm formation and filamentation of *Candida* via secreted metabolites or direct contact	[105,115,116]
Histamine-mediated immune modulation	*L. reuteri* ATCC PTA 6475	Enhances epithelial resistance to *Candida* and reduces inflammation	[117]
Cytokine-mediated immune modulation	*Saccharomyces boulardii*	Suppresses pro-inflammatory cytokines, such as IL-8 and IL-1β, whilst enhancing anti-inflammatory cytokines, such as IL-4 and IL-10. Reduces *Candida*-associated inflammation and supports epithelial integrity	[118]

## 4. Evidence of Probiotic Effects on *Candida*

*Candida* biofilms are notoriously resilient, protecting fungal cells from the host immune system and antifungal drugs. Some probiotics have, however, shown anti-biofilm activity that could be harnessed to aid treatment of candidiasis. *L. reuteri* produces a range of immunomodulatory and antimicrobial substances to protect the host from infection, such as hydrogen peroxide, which signals immune cells to reinforce epithelial defences, especially in the gut [101,119]. Hydrogen peroxide alters cysteine residues on key signalling molecules to activate pathways such as MAPK, EGFR, and NF-κB, in turn regulating cytokine production and cell migration [102,120]. *L. reuteri* is a particularly safe probiotic, having been extensively tested for its safety in adults, infants, children, and even HIV-positive individuals [121,122,123]. A dose as high as 2.9 × 10^9^ colony-forming units per day was well tolerated, safe, and efficacious in humans [124]. *L. reuteri* is also noted to produce reuterin, a broad-spectrum antimicrobial that can inhibit bacteria, protozoa, and fungi, such as *Candida* [125]. It has long been thought that the principle antimicrobial agent in reuterin is 3-hydroxypropionaldehyde (3-HPA), responsible for much of its fungicidal activity through oxidative stress induction and enzymatic interference [109]. However, recent evidence suggests that acrolein (of which 3-HPA is its precursor) may be the more potent agent [110]. It is important to note that acrolein has been shown to react with creatinine to yield the products 2-amino-3-methylimidazo(4,5-f)quinolone (IQ) and 2-amino-3,8-dimethylimidazo[4,5-f]quinoxaline (MeIQx), which are recognised as group 2A (probable carcinogen) and group 2B (possible carcinogen), respectively, by the International Agency for Research on Cancer [126]. However, reuterin production varies significantly between strains. One study tested four strains of *L. reuteri* ATCC PTA: 6475, 5289, 55730, and CF48-3A. Planktonic cultures produced 2.32, 2.3, 31.89, and 36.24 mmol reuterin/10^12^ cells, respectively [111]. *L. reuteri* ATCC PTA 6475 also releases histamine to complement its antimicrobial activity by enhancing epithelial resistance and reducing inflammatory stress that may otherwise disrupt probiotic colonisation [117].

*Lactobacillus acidophilus*, *Lactiplantibacillus plantarum L. reuteri*, and *L. rhamnosus* have all been found to inhibit several virulence factors of *Candida* in vaginal infections, most notably by *C. parapsilosis*. Fungal viability was significantly reduced after 24 h of co-incubation with cell-free supernatants of *L. acidophilus*, *L. plantarum*, and *L. rhamnosus.* These strains also significantly inhibited adhesion of *C. parapsilosis* to the vaginal epithelium [127]. An in vitro study by Vilela et al., 2015 [115] demonstrated the inhibitory effects of *L. acidophilus* ATCC 4356 on biofilm formation and filamentation of *C. albicans*. It was determined that the 24 h growth phase of *L. acidophilus* was optimal, with a 57.52% inhibition of *C. albicans*. To discern between direct cell contact and soluble factors, the culture filtrate at 24 h was also co-cultured with *C. albicans*, and a 45.10% reduction of cells was observed. Quantitative analysis of filamentation showed that both cell contact and culture filtrate inhibited the number of hyphae produced, implying that *L. acidophilus* ATCC 4356 secretes substances with anti-*Candida* properties. The study proceeded to elucidate these findings in an in vivo model using the invertebrate *Galleria mellonella*. The control groups consisted of larvae infected with *C. albicans* and treated with either PBS or MRS broth. A total of 100% of the control larvae died within 48 h. Contrastingly, larvae infected with *C. albicans* that were injected with *L. acidophilus* displayed a 20% survival rate. This survival rate of *G. mellonella* was maintained in both the group treated with *L. acidophilus* prophylactically and the group treated with *L. acidophilus* therapeutically, providing strong evidence that probiotic intervention with *L. acidophilus* could serve as a viable strategy to mitigate fungal virulence in vivo [115].

Certain probiotics secrete antimicrobial metabolites in order to supress competing microbes [128]. This biochemical interference can be particularly useful in limiting opportunistic infections such as candidiasis. Key antimicrobial metabolites consist of organic acids, hydrogen peroxide, bacteriocins, and short chain fatty acids [129]. Many probiotics, including *L. acidophilus*, *L. rhamnosus*, *B. bifidum*, and *Leuconostoc mesenteroides* (found naturally in fermented foods, such as kimchi and sauerkraut, as well as a variety of fruits and vegetables), produce lactic acid, lowering the pH and creating an acidic environment that inhibits *Candida* growth and filamentation. When *Candida* grows in an acidic environment (such as the vagina), its cell wall undergoes remodelling, exposing β-glucan and chitin [103]. This ‘unmasking’ of *Candida* enhances innate immune recognition and ultimately leads to its elimination. *Lactobacillus* spp. are classified as lactic acid bacteria (LAB) due to their production of lactic acid as a byproduct of sugar fermentation [130]. Unlike *Candida*, LAB are resistant to the acidic environment they create. *Lactobacillus* spp. contain F_0_F_1_-ATPase, which establishes proton pumps across the membrane, thought to be responsible for their unusually high resistance to acidic conditions [131]. *Bacillus caldontenax* upregulates repair enzymes to protect macromolecules from acid-induced damage [132]. *Candida* is evidenced to autoinduce the transition from yeast to hyphal form by raising the extracellular pH when it is acidic. It does this by metabolising amino acids to release ammonia, thus alkalinising the extracellular pH and allowing the transition to hyphal form. The acidic pH brought about by LAB, therefore, suppresses some of *Candida*’s virulence traits, making them promising candidates for probiotic interventions that counteract *Candida* pathogenicity [133].

A recent in vitro study co-cultured *C. albicans* with faecal microbiota from six healthy individuals. It was determined via 16S rRNA gene amplicon profiling that *Bifidobacterium adolescentis* was the strain of probiotic most correlated to the antagonistic activity observed against *Candida*. Culture supernatants of *B. adolescentis* inhibited *C. albicans* in vitro. Acetate and lactate found in the culture supernatant were important contributors to the inhibitory activity of *B. adolescentis*. Increasing the pH of the bacterial supernatant and fermentation acids caused a reduction in their anti-*Candida* properties, indicating that the fermentation acids alone are not responsible for the inhibition of *Candida* but more the acidic pH they produce [134].

LAB are the predominant producers of biosurfactants, although not all LAB are capable of this. Examples of biosurfactant producing LAB are *Limosilactobacillus fermentum*, *L. acidophilus*, *Lactobacillus paracasei*, *L. plantarum*, *Lactococcus lactis*, *Streptococcus thermophilus*, and *Propionibacterium freudenreichii* [104]. Biosurfactants can generally be divided into protein–carbohydrate complexes, lipids, or fatty acids. One study revealed *L. fermentum* B54 could produce up to 93 mg of biosurfactant per gram of cell dry weight (CDW), and *L. acidophilus* RC14 could produce 88 mg per gram CDW. The impact of these biosurfactants on the early adhesion of *Enterococcus faecalis* 1131 was assessed using a parallel-plate flow chamber with glass surfaces, either untreated or coated with biosurfactant layers. Initial deposition rates were derived from the linear movement trajectories, revealing that biosurfactants produced by *L. acidophilus* RC14 and *L. fermentum* B54 reduced the deposition rate of *E. faecalis* 1131 by 76% and 65%, respectively. After a 4 h incubation period, the number of adherent cells also decreased by 82% and 72%, respectively. These early findings highlight the powerful antimicrobial interference brought about by biosurfactants [135]. This principle was later tested on *Candida*. Biosurfactants produced by *Levilactobacillus brevis* CV8LAC were applied to medical-grade silicone disks to assess their efficacy in preventing *Candida* adhesion and biofilm formation. Co-incubation of *C. albicans* with 2000 μg mL^−1^ biosurfactant markedly reduced biofilm formation, with inhibition rates of 89%, 90%, and 90% at 24, 48, and 72 h. Pre-coating the disks inhibited adhesion and biofilm formation by 62%, 53%, 50%, and 43% at 1.5, 24, 48, and 72 h. These findings imply that *L. brevis* CV8LAC biosurfactants significantly reduce the ability of *C. albicans* to adhere to medical-grade silicone and, hence, form biofilms, though pre-coating is less effective than co-culturing. Since medical-grade silicone can only be treated prior to implantation, realistic prevention rates are closer to 43–62%, highlighting the need for more dynamic or sustained delivery methods to improve in vivo outcomes [105].

The prevention of *Candida*’s adhesion to host tissues and biofilm formation has been further explored through in vivo studies. *Saccharomyces cerevisiae* KTP strain and *Issatchenkia occidentalis* apple cider (ApC) have been tested on five non-*albicans* strains—*C. tropicalis*, *C. krusei*, *C. glabrata*, *C. parapsilosis*, and *C. auris*—on the nematode *Caenorhabditis elegans*. When the probiotics *S. cerevisiae* KTP and *I. occidentalis* ApC were co-incubated with the *Candida* strains, adhesion of *C. krusei*, *C. glabrata*, and *C. parapsilosis* was reduced by 43–52%, and adhesion of *C. tropicalis* was reduced by 33–42%. Importantly, heat-killed probiotics were exposed to non-*albicans* strains in order to determine whether metabolic activity is crucial for a functional probiotic. The results indicated that heat-killed probiotics were rendered inactive and failed to inhibit adhesion of *Candida* to abiotic surfaces. These findings highlight that adhesion inhibition is not solely a product of probiotic presence but requires metabolically active cells at effective concentrations to exert meaningful antifungal effects. This has significant implications on formulation strategies and clinical outcomes, as probiotic treatments must retain their viability and therapeutic efficacy within the host [136].

Many probiotics utilise bacteriocins, such as nisin and plantaricin, to inhibit competing microbes and enhance their survival within the microbiome. *L. lactis* is known to produce the class I bacteriocin nisin, a lantibiotic [112]. Nisin exhibits potent antibacterial activity against Gram-positive bacteria, such as *Enterococci*, *Staphylococci*, *Streptococci*, *Bacillus cereus*, and *Listeria monocytogenes*, by binding to lipid II, forming membrane pores and halting cell wall synthesis [113,137]. Its antifungal effects are more indirect, often relying on synergistic interactions that enhance membrane permeability or inhibit biofilm formation in species such as *Candida*. A recent study demonstrated that nisin A lowered the minimum inhibitory concentration (MIC) of amphotericin, miconazole, and micafungin against *C. albicans*, *C. glabrata*, *C tropicalis*, and *C. parapsilosis* [114]. In another study, nisin inhibited azole-resistant *C. tropicalis* growth, showing a four-fold reduction in OD_600nm_ values at the 8 h incubation time point. Crystal violet staining was used for biofilm quantification, revealing a significant decrease in OD_570nm_ values. The results indicate that nisin exhibits potent antifungal and anti-biofilm effects against clinical isolates of azole-resistant *C. tropicalis*, highlighting its potential as an alternative therapeutic agent for treating infections caused by azole-resistant *C. tropicalis* [116].

The short chain fatty acid butyrate is a metabolite produced by various anaerobic bacteria, particularly in the colon, through fermentation of dietary fibres and starches [138]. *C. butyricum* is a symbiotic probiotic frequently used in China, Japan, and South Korea, embraced for its ability to suppress bacterial infections such as *Clostridioides difficile* and support gut barrier integrity [139]. The strain CBM 588 is particularly well studied and has been part of the MIYA-BM probiotic formulation in Japan for over 40 years since its development in the early 1970s [140]. It was selected due to its ability to produce high amounts of butyric acid and its strong safety profile [106]. CBM 588 was shown to lack any pathogenicity factors and the genes necessary for production of clostridial toxin [141]. In a rat model of dextran sodium sulphate-induced colitis, *C. butyricum* CBM 588 notably increased levels of butyric acid in the cecum, indicating that it actively produces high levels of butyric acid in vivo [142]. Many *Roseburia* spp. produce butyrate; however, due to their anaerobic nature, they are proving difficult to harness in commercial probiotic formulations, leading to their label as a “next generation probiotic” [143]. However, *Roseburia intestinalis* has shown no toxicity in animal models and adheres to epithelial cells without disrupting microbiota diversity [144]. Butyrate is the preferred energy source for epithelial cells, meeting 70–80% of colonocyte energy requirements [107]. Butyrate also enhances barrier integrity by promoting tight junction stability, defending against pathogenic microbes such as *Candida* [108]. This makes *R. intestinalis* a potentially promising probiotic if a suitable method of anaerobic delivery can be established.

Probiotics inhibit *Candida* not only through adhesion interference, biofilm disruption, and antimicrobial metabolite production but also via the deceptively simple yet effective mechanism of competitive inhibition [145]. Probiotic colonisation creates nutrient scarcity for competing pathogenic microbes, making it difficult for pathogenic infection to take hold or thrive [146]. Organic acids, such as lactic, acetic, and propionic, produced by many probiotics lower pH conditions to an acidic environment, inhibiting many opportunistic pathogens such as *Candida* [147]. *Saccharomyces boulardii* is a probiotic yeast known for its protective effect against numerous enteropathies [148]. One in vitro study of the human epithelial cell lines Caco-2 and Intestin 407 revealed that *S. boulardii* significantly inhibited adhesion of *C. albicans* to epithelial cells [149]. Many probiotics are additionally capable of modulating the pathogen or host immune response in order to mitigate infection [150]. The aforementioned study concluded that *S. boulardii* was capable of suppressing the IL-8 gene expression of *C. albicans*, in turn reducing the hosts cytokine-mediated inflammatory response [149]. In another in vitro study, intraepithelial lymphocytes (IELs) were infected with *E. coli* and *C. albicans*, and cytokine levels were measured by an immunosorbent assay. Secretion of pro-inflammatory cytokines, such as IL-1β, was reduced in IELs incubated with *S. boulardii*, whereas anti-inflammatory cytokines, such as IL-4 and IL-10, saw an increase in secretion. Therefore, *S. boulardii* may have a protective effect on host epithelia by modulating the immune response during pathogenic infection by *C. albicans* [118].

## 5. Clinical Trial Evidence of Probiotic Effects on *Candida* spp.

Table 2 summarises clinical studies evaluating the antifungal efficacy of various probiotic strains against *Candida* species in the gastrointestinal tract, oral cavity, and vagina. The studies encompass diverse probiotic formulations and anatomical sites, illustrating the variability in probiotic approaches and the complex dynamics of host–microbe interactions in distinct bodily environments. Across the selected clinical trials, participants included distinct groups such as the elderly, women, children, and preterm neonates. The selected studies were included based on their variation in strain number, formulation complexity, and the variety of participant populations evaluated [151,152,153,154,155,156,157,158,159,160,161]. Comparative analysis across mucosal sites of *Candida* infection highlights the importance of site-specific probiotic interventions, as the antifungal efficacy of a formulation may be pronounced at one anatomical site but insignificant at another.

Across the selected studies, the formulations consisted of single- and multi-strain formulas of *Saccharomyces*, *Lactobacillus*, *Bifidobacterium*, and *Propionibacterium*. Probiotic administration was predominantly oral due to its simplicity and high participant adherence. In studies targeting vaginal *Candida* infections, intravaginal delivery via pessaries or globules was employed to enable rapid localised action. Trial durations ranged from a matter of days to months, allowing an understanding of the duration that probiotics may require to exert full efficacy. Treatment was not isolated to those with current *Candida* infections; the groups tested also encompassed individuals highly susceptible to candidiasis, such as vulnerable preterm neonates and elderly denture users. One additional study (not included in Table 2 due to the small sample size) investigated the effect of *Bifidobacterium* and *Lactobacillus* on 24 women, 17 of which were living with HIV [162]. All participants consumed DanActive^TM^ yoghurt once daily for 15 days and then YoPlus^TM^ yoghurt for 15 days, with a 30-day washout period in between. In the women with HIV, fungal colonisation was 54% at non-intervention periods but was reduced significantly to 29% following consumption of DanActive^TM^ yoghurt. The findings suggest that probiotics may have a protective effect for immunocompromised individuals against fungal infection. Safeguarding those most vulnerable to candidiasis, such as those with HIV, preterm neonates, or elderly denture wearers, is a critical component of preventative healthcare.

Outcome measures of the studies generally focused on reducing *Candida* colonisation levels, measured using viable cell enumeration to provide quantitative data. Mendonça et al., 2012 [155] further included salivary IgA quantification to determine the mucosal immune response. Roy et al., 2014 [153] included Platelia *Candida* ELISA, a method used to detect mannan antigen and anti-mannan bodies, to evaluate the risk of systemic candidiasis. Outcome measures also involved an assessment of symptom reduction, particularly in vaginal infections. Standardised scoring systems and patient-reported outcomes were used to measure symptoms such as erythema, itching, discomfort, and discharge. These dual-focus endpoints provide a better understanding of how microbial shifts can correlate with tangible clinical improvement. Most trials used double-blinded randomised controlled trials (RCTs) in order to safeguard against bias of any kind and ensure the reliability and validity of findings. Such rigour is especially important in studies of microbiome–host interaction, as outcomes could be influenced by patients’ subjective symptom reporting. Where multiple endpoints are assessed, maintaining interpretive precision is critical to avoid misattribution and analytical confounding.

The studies by Demirel et al., 2013 [151] and Mendonça et al., 2012 [155] are outliers as they were not double-blinded RCTs. Demirel et al., 2013 [151] used a randomised comparative study, perhaps due to ethical concerns. Withholding this potentially beneficial treatment from a control group of vulnerable preterm neonates may have raised ethical concerns. Moreover, blinding would have been challenging given the differing formulations: *S. boulardii* was powdered, whereas nystatin was in an oral suspension. The benefit of double blinding the trial is debatable, as the neonates cannot symptom report and this was not an outcome being measured. Methodological pragmatism in this case reflected a deliberate balance between ethical safeguards and trial feasibility. Newer research into *S. boulardii* has highlighted the strain CNCM I-745 as a potential probiotic treatment for acute gastroenteritis and antibiotic-associated diarrhoea. Clinically, this strain has outperformed *L. rhamnosus* GG and *Bacillus clausii* and shown a favourable safety profile [163].

Mendonça et al., 2012 [155] used a single-arm, pre-post intervention study. Yakult^®^ is widely available and has a well-established safety profile, meaning the risk to elderly trial participants is minimal but potential benefits are high. Withholding the treatment from the vulnerable older population would raise ethical red flags. The trial design allowed researchers to study the effects of probiotics within the same subjects over time rather than against a control group. This approach may offer insight into the variability in individuals’ response to Yakult^®^. The study design prioritised feasibility and participant welfare over definitive causal inference. Although the two studies that did not employ a double-blinded RCT design may be subject to methodological limitations, such as bias and reduced validity, they nonetheless provide valuable preliminary data. Collectively, all studies contribute meaningful evidence supporting the potential benefits of probiotics, particularly in the context of *Candida* colonisation at multiple mucosal sites around the body.

A 2025 meta-analysis of 13 RCTs determined an odds ratio of 0.38, suggesting that probiotics have a significant protective effect against oral candidiasis [164]. However, the I^2^ index of this statistic is 60.3%, meaning substantial heterogeneity between studies. When focussing on individuals with candidiasis or related diseases, the odds ratio was 0.40, with an I^2^ index of 18.2%, implying greater consistency in these findings and more reliable evidence for probiotic efficacy in individuals already affected by candidiasis, strengthening the case for targeted probiotic interventions in this group. Another meta-analysis of 12 English and Chinese RCTs calculated an odds ratio of 7.80, indicating a strong association between probiotics and successful treatment of oral candidiasis [165]. Recurrence rates had an odds ratio of 0.06, suggesting probiotics not only eliminated *Candida* but prevented its re-colonisation. This exceptionally low odds ratio reflects a strong protective effect, indicating that probiotic use may confer sustained benefits in reducing relapse.

## 6. Challenges and Limitations in Probiotic Management of Candidiasis

One of the main challenges in using probiotics in prophylaxis or treatment of candidiasis is the variability of individuals’ baseline microbiome composition. This complicates probiotic colonisation and functional efficacy, as strain engraftment and metabolite production are dependent on the host’s existing microbial ecosystem [166]. In individuals with a microbiome not dominated by *Lactobacillus*, probiotic colonisation may be particularly difficult to establish and sustain. The reduced presence of LAB can elevate the pH, creating an environment less hospitable to acidophilic strains such as *Lactobacillus*, which are commonly included in probiotic formulations [167]. Alkalinisation of the gut or vaginal tract can lead to overgrowth of opportunistic pathogens such as *Candida* [168]. The composition of the microbiome not only varies between individuals but can fluctuate with diet, antibiotic usage, ethnicity, and geographical location [169,170]. Western style diets are high in sugar, fat, and processed foods, tending to favour *Proteobacteria* species [171,172]. These often dominate via competitive inhibition and reduce overall microbial diversity. Low dietary fibre intake reduces populations of beneficial Firmicutes, such as *Eubacterium* and *Roseburia*, impairing the production of short chain fatty acids that are critical for the integrity of the gut barrier and immune regulation [173]. Reduced microbial diversity disrupts cross-feeding networks, whereby metabolic products from one species serve as substrates for another, further weakening ecosystem resilience and host protection [174].

In immunocompromised individuals, weakened mucosal defences may in rare cases allow probiotics to translocate through the epithelium and cause systemic infection, such as bacteraemia or fungaemia [175]. This risk is highest in those with central venous catheters, mucosal damage, or neutropenia [176]. Reduced function of dendritic cells, macrophages, and neutrophils can allow even commensal probiotics to become opportunistically pathogenic [177]. In healthy individuals, *Lactobacillus* is contained in the gut [178]. However, in studies of immunocompromised patients, it has been isolated from blood cultures, suggesting a translocation through weak barriers and poor regulation by immune cells, allowing systemic infection [179,180]. Although rare, the ability of probiotics to translocate into systemic circulation reinforces the need for cautious clinical evaluation and judgement, particularly in severely immunocompromised patients.

Another key challenge in reaching a confident consensus on the effect of probiotics on *Candida* and other infections is the inconsistencies in research methodologies. Specific probiotic strains tested vary between studies, offering a broad selection of data but limiting the ability to focus in-depth on promising strains. Doses of probiotics administered can range from 10^7^ to 10^11^ colony forming units (CFU), meaning observed effects vary considerably between studies, dependent on the quantity of probiotic given [181]. The dose required to achieve therapeutic efficacy likely varies between individuals, influenced by body weight, sex, comorbidities, and baseline microbiome composition [182]. Determining the optimal therapeutic range of a probiotic formulation is, therefore, complex and requires further research. Dosage requirements also depend on the method of delivery, as oral formulations must be able to withstand the acidic conditions of the stomach to ensure that the probiotics remain metabolically active at their target tissue [183]. Local delivery via vaginal suppository bypasses gastrointestinal degradation to allow direct action on the vaginal mucosa at the site of infection, potentially enhancing strain-specific efficacy [184].

The duration of in vitro, in vivo, and clinical trial research periods often varies significantly, with some trials lasting just a week and others extending over several months [152,154]. The results from these different trial designs are, therefore, difficult to compare due to the potential influence of many confounding variables. In addition, end points are not standardised across studies. Whilst the majority measure for *Candida* reduction, some also evaluate clinical symptomatology [159,160] and serum immunoglobulin concentration [155]. The techniques used to measure these end points are non-uniform, often dictated by resource availability, time constraints, and funding limitations. These factors highlight the difficulties of cross-study comparisons and meta-analyses. A proportion of studies are underpowered and lack rigorous controls, resulting in inconsistent findings and reduced reliability. In order to move towards formulating clinically effective probiotic therapies for candidiasis, future research should incorporate stratified study designs with standardised protocols to enhance reproducibility.

Not all probiotics are generally recognised as safe (GRAS) by the FDA [185]. Although rare, a few cases of bacteraemia and fungaemia have been reported in association with probiotic use, primarily in immunocompromised individuals [175]. Great care must be taken when exposing this subset of the population to probiotic treatment. The categorisation of probiotics falls interchangeably between food and drug categories, dependent on jurisdiction and intended use [186]. This ambiguity causes variability in safety standards and quality control of products. The viability of strains can be influenced by processing, such as compression and freeze-drying, as they can compromise cell integrity [187]. To preserve the efficacy of products, CFU counts must be validated at several points in the production process in order to maintain the functionality of the probiotics for their entire shelf life [188]. Packaging is usually designed to be oxygen and water impermeable to prevent degradation of the product; however, poor transportation or storage at incorrect temperatures by retailers may compromise the functionality or safety of the probiotic [189]. Another critical element of safe probiotic production is managing contamination risk through stringent raw material screening and end-product testing. It is vital that strain identities are confirmed and any cross-contamination is identified using high sensitivity techniques such as qPCR and MALDI-TOF [190]. Antibiotic resistance is not exclusive to pathogenic microbes. Certain probiotic strains possess antibiotic resistance genes, posing a risk of horizontal gene transfer [191]. These strains must be identified and excluded from probiotic formulations through targeted genomic screening. Complying with regulatory standards, such as those set by the FDA, is fundamental to ensuring probiotic safety, particularly as their clinical applications expand.

While probiotics appear to have promising anti-*Candida* capabilities, their functional clinical application proves more challenging due to host variability, methodological inconsistencies, and regulatory gaps. The transition from bench to bedside will require standardised and coordinated clinical trials, robust safety frameworks, and, perhaps, a personalised medicine approach.

## 7. Future Directions and Therapeutic Potential

Though select probiotics have been evidenced to confer many health benefits as standalone interventions, synergistic effects have been observed when in combination with prebiotics [192]. Prebiotics are non-digestible components of food, such as fibres and oligosaccharides, that act as nutrients for commensal bacteria or probiotics to grow and thrive, enhancing their survival and therapeutic efficacy [35,193]. Prebiotics may be provided naturally by foods such as onion, garlic, asparagus, oats, and wheat. The most widely studied prebiotics include fructooligosaccharides (FOS), isomalto-oligosaccharides (IMO), and xylooligosaccharides (XOS) [194]. Inulin, the naturally occurring fructan derived primarily from chicory root, has been proven to stimulate growth of many *Bifidobacterium* and *Bacteroides* species, amongst others [195,196,197]. IMOs are found in fermented foods, such as soy sauce, miso, and honey, and promote the growth of *Bifidobacterium*, *Lactobacillus*, and *Bacteroides* [198]. XOS are emerging prebiotics derived from certain fruits, vegetables, milk, bamboo shoots, and honey [199]. They have been reportedly utilised by *B. adolescentis* and *L. brevis*, though further strain-specific evaluations are ongoing [200]. The synergistic combination of prebiotics and probiotics is aptly named synbiotics and is increasingly being utilised in commercial probiotic formulations to enhance viability and functional efficacy [201]. Postbiotics include metabolites such as lactic acid, bacteriocins, short chain fatty acids (butyrate, acetate, propionate), amino acids, enzymes, and vitamins [202]. Future commercial formulations may integrate prebiotics, probiotics, and postbiotics in order to maximise viable CFUs reaching target sites, therapeutic efficacy, and overall host health.

As previously outlined, probiotic genera such as *Lactobacillus*, *Lactococcus*, *Limosilactobacillus*, *Streptococcus*, and *Propionibacterium* can produce biosurfactants and organic acids that weaken fungal biofilms [203,204]. This activity presents a key opportunity to enhance the penetrative efficacy of antifungal drugs through probiotic coadministration. Most existing studies either compare probiotic efficacy to that of antifungal drugs or suggest adjunctive potential. However, there remains a clear lack of combination trials to evaluate whether probiotics can modulate antifungal pharmacodynamics and clinical outcomes. Future research should prioritise mechanistic studies into probiotic–antifungal coadministration, with an attention to strain specificity. This is not without challenges—pharmacokinetic interactions between antifungal drugs and the microbiome often result in an altered microbiome composition, having the potential to limit probiotic viability and therapeutic consistency. A variety of emerging technologies may be key in addressing such complexities. Artificial intelligence-driven strain selection and synthetic organoid models offer promising approaches for evaluating probiotic–antifungal interactions under controlled conditions [182]. These systems would allow safe and reproducible testing of synergistic interactions between probiotics and existing antifungal drugs, representing a possible future direction in rational design for probiotic studies.

Another potential future direction in the field is personalised probiotic therapy, whereby individuals undergo microbiome sequencing (via stool samples) to determine their baseline microbiome composition [205]. This profile guides the selection of probiotic strains most suitable for the individual, recognising that one strain does not work equally in all members of the population. Personalised therapy not only enhances probiotic efficacy for the host but enables specific therapeutic goals to be met, such as reducing inflammation, improving constipation or diarrhoea, and even managing metabolic disorders [206]. For those susceptible to candidiasis or with recurrent infections, personalised anti-*Candida* probiotic formulations may be particularly helpful in preventing colonisation, relieving symptoms, and improving quality of life. Next generation probiotics (NGPs) are an emerging arm of precision medicine, utilising novel commensal bacteria such as *Faecalibacterium prausnitzii* and *Akkermansia muciniphila* with highly specialised functions [207]. The anti-*Candida* status of such NGPs is currently uncertain and warrants further study, presenting an exciting new avenue for future research.

## 8. Conclusions

*Candida* infections represent a growing global health concern due to increasing antifungal resistance and the emergence of non-*albicans* species. Current antifungal drugs are limited and frequently undermined by biofilm formation, efflux pumps, and ever-evolving resistance mutations. This review outlined the variety of mechanisms utilised by *Candida* to transition from commensal to pathogenic and exert dominance amongst other commensals. *Candida* is able to evade detection by the immune system and switch from yeast to hyphal form in order to form thick biofilms and colonies resilient to antifungal drugs. This review examined the role of probiotics as a novel or adjunct therapy for such infections, ranging from simple competitive exclusion to complex cellular mechanisms. The ability of *Candida* to form biofilms on host surfaces and implanted medical devices threatens those most vulnerable and immunocompromised, emphasising the need for a robust and diverse microbiome. In vitro and in vivo studies have demonstrated that probiotic strains such as *L. reuteri*, *L. acidophilus*, and *S. boulardii* can stimulate rich commensal microbe colonies and reinforce the gut barrier to protect from infection. Many probiotics have also been extensively proven to inhibit *Candida* growth and phenotypic switching, modulating *Candida*’s opportunistic nature. The range of anti-*Candida* activity observed throughout probiotic studies from simple competitive inhibition to advanced quorum sensing inhibition highlights the huge potential probiotics possess for anti-*Candida* therapy.

Clinical trials and meta-analyses suggest probiotics can reduce *Candida* colonisation in the oral cavity, gastrointestinal tract, and vagina as well as reduce the rate of recurrence, particularly in vulnerable populations. However, it was identified that heterogeneity in study design and strain selection does limit the generalised conclusions that can be drawn. Functional therapeutic efficacy is further complicated by host variability in baseline microbiome composition and immune health, prompting the need for future research into personalised probiotic approaches. Preliminary studies into coadministration of prebiotics and probiotics (synbiotics) have suggested an increase in anti-*Candida* properties, thought to be due to the production of metabolic byproducts (postbiotics). These include short chain fatty acids such as butyrate, lactic acid, and bacteriocins that have been described in this review for their strong anti-biofilm and anti-*Candida* properties. Probiotics owe much of their celebrated antimicrobial effects to prebiotics and postbiotics, implying that commercial formulations in the future may wish to incorporate all of these to enhance efficacy and results. Future research should capitalise on the understanding that postbiotic metabolites are key mediators of the positive impacts attributed to many probiotic strains. Isolation and characterisation of compounds such as butyrate, bacteriocins, lactic acid, and biosurfactants from the probiotic strains may allow more targeted research and provide specific insights into their efficacy against candidiasis and similar infections. Employing such metabolites as alternatives to administering live strains would alleviate the risk of bacteraemia and fungaemia in critically ill and immunocompromised patients as well as reduce the variability in therapeutic outcomes associated with strain-specific viability. A potential new avenue for antifungal research could be to consider pairing probiotics or postbiotic metabolites with traditional antifungal drugs to determine whether they can increase antifungal efficacy and clinical outcomes for patients. Utilising current technologies such as artificial intelligence software to screen strains for their potential in preliminary studies will allow the field to advance its understanding of the scope of probiotic commercial applications.

To summarise, probiotics have proven to be effective in reducing *Candida* colonisation and reducing symptoms of candidiasis at numerous sites in the human body. Some have even demonstrated a protective effect on vulnerable immunocompromised or elderly populations. Incorporating prebiotics and postbiotics into commercial formulations may enhance therapeutic efficacy and even reduce the likelihood of recurrence. While current evidence supports their adjunctive potential, rigorous stratified research is needed to translate these findings into safe and effective clinical applications. Bridging the gap between laboratory and store shelf requires rigorously designed clinical trials, scalable strain-specific production, and regulatory clarity.

## Figures and Tables

**Table 2:** Summary of clinical trials evaluating probiotics against *Candida* spp. at various anatomical sites.

Reference	Probiotic(s)	Pathogen(s)	Methodology	Results	Summary
Gastrointestinal tract					
[151]	*S. boulardii*	*Candida* spp.	Prospective randomised comparative study 181 preterm neonates (gestational age ≤ 32 weeks) with birth weight ≤ 1500 g Breast milk or formula supplemented with *S. boulardii* (*n* = 91) or nystatin suspension (*n* = 90) every 8 h Skin, stool, and rectal samples collected to detect for *Candida* Blood, urine, and CSF samples collected to detect invasive infection	Colonisation of skin and stool was similar in *S. boulardii* and nystatin groups Cases of sepsis were significantly lower in *S. boulardii* group	*S. boulardii* and nystatin are similarly effective at reducing *Candida* infection in preterm neonates; however, *S. boulardii* is significantly better at preventing septic infection
[152]	*L. acidophilus*, *L. rhamnosus*, *B. bifidum*, *S. boulardii*, *S*. *thermophilus*, *Bifidobacterium longum*	*Candida* spp.	Prospective double-blinded randomised controlled trial (RCT) 150 children (aged 3 mo–12 y) on broad-spectrum antibiotics for at least 48 h Probiotic (*n* = 75) or placebo (*n* = 75) given twice a day for 7 days Urine and blood samples and rectal swab collected to quantify *Candida*	On day 7, 27.9% of probiotic group and 42.6% of placebo group were colonised, showing significant reduction in GI tract candidiasis Probiotic group had significantly lower cases of candiduria Cases of candidaemia did not differ significantly	Probiotics may reduce *Candida* infections in the GI tract or urogenital tract of children taking broad-spectrum antibiotics but show no evidence of reducing candidaemia infections
[153]	*L. acidophilus*, *B. lactis*, *B. longum*, *B. bifidum*	*C. albicans* *C. glabrata* *C. krusei* *C. parapsilosis*	Prospective double-blinded RCT 112 preterm neonates (gestational age ≤ 37 weeks) with birth weight < 2500 g Breast milk supplemented with probiotic mixture (*n* = 56) given daily from first 72 h for 6 weeks or until discharged or breast milk with placebo (*n* = 56) under the same conditions Gastric aspirate and stool samples collected to detect *Candida* Blood samples and platelia *Candida* test collected to detect invasive infection	Hospitalisation time reduced in probiotic group *Candida* in stool reduced in probiotic group Full enteral feed establishment was earlier in probiotic group	Probiotics can reduce *Candida* colonisation in the GI tract of preterm neonates and hasten progression to full enteral feeding
Oral cavity					
[154]	*L. rhamnosus* GG (ATCC 53103), *L. rhamnosus* LC705, *P. freudenreichii* ssp. *shermanii* JS	*C. albicans* *C. glabrata* *C. krusei* *C. tropicalis*	Prospective double-blinded RCT 276 elderly people 50 g of probiotic (*n* = 136) or control (*n* = 140) cheese eaten daily for 16 weeks Saliva samples collected to detect and quantify *Candida*	*Candida* in saliva of probiotic group decreased from 30.4% to 20.7% *Candida* in saliva of control group increased from 28.0% to 34.0% Probiotics reduced the risk of a high yeast count by 75.0%	Probiotics help lower *Candida* levels in the oral cavity of elderly people and alleviate hyposalivation, oral lesions, and pain
[155]	*Lactobacillus casei*, *Bifidobacterium breve* (Yakult LB^®^)	*C. albicans* *C. tropicalis* *Candida guilliermondii* *C. glabrata* *Candida lipolytica* *C. krusei* *Candida kefyr* *C. parapsilosis*	Single-arm, pre-post intervention study 42 women aged ≥ 65 y All women consumed a bottle of Yakult LB^®^ 3 times per week for 30 days Saliva samples collected for identification of *Candida* species and IgA quantification	Probiotic therapy significantly reduced the prevalence of *Candida* from 92.9% to 85.7% *Candida* was completely eradicated in 11.9% of participants There was a significant increase in anti-*Candida* IgA levels	Yakult LB^®^ effectively reduced *Candida* levels in elderly women, with the ability to eradicate its colonisation entirely partly due to increasing IgA levels
[156]	*L. rhamnosus* HS111, *L. acidophilus* HS101, *B. bifidum*	*Candida* spp.	Prospective double-blinded RCT 59 (6.8% attrition rate) denture wearers harbouring *Candida* but with no clinical symptoms of oral candidiasis Probiotic group (*n* = 30) placed probiotic capsule on dentures daily for 5 weeks Placebo group (*n* = 29) performed same regimen with placebo capsule Palatal swab collected to quantify and identify *Candida* species	*Candida* spp. detected in 92.0% of placebo group and 16.7% of probiotic group	Probiotic-treated dentures can reduce *Candida* colonisation of the mouth by 75.3%. Results are independent of initial *Candida* level, denture age, or colonising species
[157]	*L. reuteri* DSM 17938, *L. reuteri* ATCC PTA 5289	*Candida* spp.	Prospective double-blinded RCT 215 (19% attrition rate) elderly people (aged 60–120 y) Probiotic group (*n* = 106) received a probiotic lozenge twice daily (morning and evening) for 12 weeks, and placebo group (*n* = 109) received a placebo lozenge under the same regimen Saliva and plaque samples collected (baseline and endpoint) to quantify *Candida* and levels of dental plaque and gingival inflammation	Statistically significant reduction in prevalence of high *Candida* counts in saliva and plaque of probiotic group but not placebo group of frail elderly patients in a nursing home No significant difference in levels of supragingival plaque or bleeding upon probing	Probiotics can significantly reduce *Candida* levels in the saliva and plaque but do not affect gingival inflammation
[158]	*L. plantarum* CCFM8724	*C. albicans*	Prospective double-blinded RCT 100 children (ages 3–6 y/o) with early childhood caries (ECC) Probiotic group (*n* = 50) received *L. plantarum* CCFM8724 daily for 28 days, followed by a 14-day washout period. Control group (*n* = 50) followed the same regimen with placebo Dental plaques were collected on days 1 and 28 and effects measured by sequencing V3-V4 region of 16S rDNA. On days 1, 14, 28, and 42, qPCR was used to quantify the effect on *C. albicans* in saliva	*L. plantarum* CCFM8724 significantly reduced the amount of *C. albicans* in the saliva of children with ECC Abundance of *Firmicutes*, *Granulicatella*, and *Gemella* increased Abundance of *Proteobacteria*, *Neisseria*, *Bifidobacterium*, and *Catonella* decreased	*L. plantarum* CCFM8724 can significantly reduce the amount of C. albicans in the saliva of children with ECC
Vagina					
[159]	*L. rhamnosus* GR-1, *L. reuteri* RC-14	*Candida* spp.	Prospective double-blinded RCT 55 women diagnosed with vulvovaginal candidiasis (VVC) All participants given single dose of fluconazole, followed by 2 tablets every morning for 4 weeks Probiotic group (*n* = 29) and placebo group (*n* = 26) Vaginal swabs collected to detect *Candida* and clinical evaluation to determine VVC status	Probiotics reduced the levels of *Candida* and significantly reduced symptoms of VVC, such as vaginal itching, burning, and discharge, as well as painful sex and urination when combined with fluconazole treatment	Probiotics reduced *Candida* colonisation and symptoms of VVC when combined with fluconazole treatment. Adverse side effects were rare and not definitively linked to the probiotic treatment
[160]	*L. acidophilus*, *L. rhamnosus*, *Lactobacillus delbrueckii* ssp. *bulgaricus*, *S. thermophilus*	*Candida* spp.	Prospective open-label RCT 436 women (aged 17–50 y) with *C. albicans* vaginal infection Control group (*n* = 207) was given a single dose of fluconazole and fenticonazole Probiotic group (*n* = 209) was given the same antifungal treatment and, on day 5 post-azole, commenced a 10-day probiotic regimen of one application of a vaginal globule per day Clinical and microbiological tests were carried out to determine infection status	Fungal clearance in the azole-only group was 93.7%, and unpleasant symptoms, such as itching and discharge, persisted in 79.7% of participants In the azole plus probiotic group, fungal clearance was 95.2%, and symptoms persisted in just 31.1% of participants	Probiotics delivered locally to the vagina can increase *Candida* clearance and significantly reduce the persistence of VVC symptoms when combined with azole treatment
[161]	*Lactobacillus crispatus* DSM32717, DSM32720, DSM32718, and DSM32716	*C. albicans* *C. glabrata*	Prospective double-blinded RCT 89 bacterial vaginosis (BV) and 93 vulvovaginal candidiasis (VVC) patients (aged 18–50 y/o) Patients from each diagnosis group received either oral or vaginal probiotic capsules or placebo capsules over a 3-month period Vaginal, intestinal, and general health monitored weekly by questionnaire Blood analyses at beginning and end of trial Vaginal samples collected monthly and microscopic and molecular analyses performed	In BV patients, both oral and vaginal capsules reduced symptoms, with a remarkable improvement in Nugent score, amount and smell of discharge, and itching/irritation. In VVC patients, both oral and vaginal capsules reduced discharge and itching/irritation	*L. crispatus* strains can significantly reduce symptoms of BV and VVC

## Data Availability

No new data were created or analyzed in this study. Data sharing is not applicable to this article.
